# Soy Isoflavones Induce Cell Death by Copper-Mediated Mechanism: Understanding Its Anticancer Properties

**DOI:** 10.3390/molecules28072925

**Published:** 2023-03-24

**Authors:** Mohd Farhan, Mohamed El Oirdi, Mohammad Aatif, Insha Nahvi, Ghazala Muteeb, Mir Waqas Alam

**Affiliations:** 1 Department of Basic Sciences, Preparatory Year Deanship, King Faisal University, Al Ahsa 31982, Saudi Arabia; 2 Department of Public Health, College of Applied Medical Sciences, King Faisal University, Al Ahsa 31982, Saudi Arabia; 3 Department of Nursing, College of Applied Medical Sciences, King Faisal University, Al Ahsa 31982, Saudi Arabia; 4 Department of Physics, College of Science, King Faisal University, Al Ahsa 31982, Saudi Arabia

**Keywords:** isoflavones, genistein, daidzein, copper, anticancer, cell death

## Abstract

Cancer incidence varies around the globe, implying a relationship between food and cancer risk. Plant polyphenols are a class of secondary metabolites that have recently attracted attention as possible anticancer agents. The subclass of polyphenols, known as isoflavones, includes genistein and daidzein, which are present in soybeans and are regarded as potent chemopreventive agents. According to epidemiological studies, those who eat soy have a lower risk of developing certain cancers. Several mechanisms for the anticancer effects of isoflavones have been proposed, but none are conclusive. We show that isoflavones suppress prostate cancer cell growth by mobilizing endogenous copper. The copper-specific chelator neocuproine decreases the apoptotic potential of isoflavones, whereas the iron and zinc chelators desferroxamine mesylate and histidine do not, confirming the role of copper. Reactive oxygen species (ROS) scavengers reduce isoflavone-induced apoptosis in these cells, implying that ROS are cell death effectors. Our research also clearly shows that isoflavones interfere with the expression of the two copper transporter genes, *CTR1* and *ATP7A*, in cancerous cells. Copper levels are widely known to be significantly raised in all malignancies, and we confirm that isoflavones can target endogenous copper, causing prooxidant signaling and, eventually, cell death. These results highlight the importance of copper dynamics within cancer cells and provide new insight into the potential of isoflavones as cancer-fighting nutraceuticals.

## 1. Introduction

Cancer is still the biggest cause of death worldwide [1]. Cancer formation and progression is a dynamic and long-term process driven by changes in genetic sequences and the acquisition of particular traits that allow for the establishment of full malignancy [2]. It has been suggested that even within a single type of tumor, various genetic variations can be found in different altered cells [3]. Furthermore, a comparable tumor in various people tends to have distinct mutations and genetic components involved. Despite the complexity of the carcinogenesis process and the various types of mutations, the arising characteristic changes are often a small number of molecular, biochemical, and cellular traits, which frequently lead to changes in the metabolic status of the tumor as compared to normal cells [4]. Consequently, targeting the emerging metabolic alterations, which are unique to all types of cancer, rather than the mutations responsible for these metabolic changes, can speed up the discovery of prospective anticancer drugs.

In recent years, there has been a lot of interest in the possibility that some diet-derived substances can prevent or postpone the initiation of cancer. It is thought that appropriate lifestyle adjustments, including food habits, might avert more than two-thirds of human cancers [5]. Polyphenols, abundant in plants, are the most common group of physiologically active secondary metabolites. Plant polyphenols are important components of the human diet, and several have anticancer properties [6]. Epidemiology studies have shown that people who eat a lot of soy have a lower chance of developing various cancers, including colon, breast, and prostate cancer [7,8]. Two major isoflavones found in a variety of soybeans and soy-based products are genistein and daidzein [9,10,11]. Both of these isoflavones have been found to exhibit anticancer properties in both in vitro and in vivo cancer models [12,13,14,15,16,17]. Despite the fact that these polyphenolic compounds have been shown to induce apoptosis in cancer cell lines, the mechanism of anticancer action, which must operate as an upstream signal, is unknown. Flavonoids [18], tannic acid and gallic acid [19], curcumin [20], gallocatechin [21], and resveratrol [22] are all known to cause oxidative DNA damage. Copper is an important redox active metal ion present in chromatin, closely associated with DNA bases and mobilized by metal chelating agents [23].

Previously, we had proposed a hypothesis according to our observations as well as similar to those of other researchers, which stated that the anticancer mechanism of plant polyphenols includes intracellular copper mobilization [24,25]. Perhaps chromatin-bound copper and the prooxidant effect initiate ROS-mediated cellular DNA breakage and consequent cell death [24,25]. Such a prooxidant mechanism results from a redox-active microenvironment in cancer cells due to elevated levels of copper [26].

Over the decades, there has been solid evidence suggesting a large elevation of serum, plasma, and intracellular copper levels in all types of malignancies [27,28,29,30,31]. Copper is required by tumor cells to increase the proliferation and migration of endothelial cells as well as required for the release of angiogenic factors by tumor cells [32]. Copper and zinc are the predominant ions in the nucleus [24], despite the fact that iron is present in significantly greater quantities in typical biological systems. In light of our findings and those of others, our hypothesis has gained considerable attention in recent years [24,25,26].

**Figure 1:**
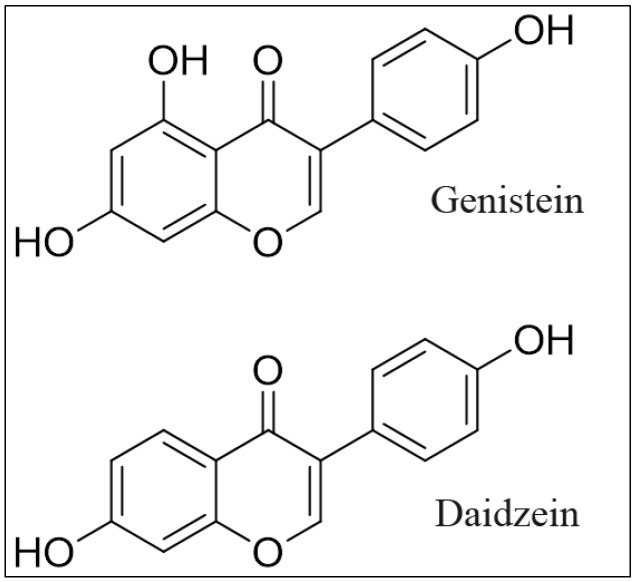
Chemical structure of genistein and daidzein.

In the present study, we confirm the mechanism of action of plant-derived polyphenolic compounds by utilizing the isoflavone family. We demonstrate that both genistein and daidzein decrease cell proliferation and induce apoptosis in prostate cancer cell lines. Such cell death is significantly inhibited by the cuprous chelator neocuproine and other ROS scavengers, including superoxide dismutase, catalase, and thiourea. Copper chelation inhibits this ROS formation, validating the conclusion that the mobilization of intracellular copper by isoflavone results in the development of ROS that induces prooxidant cell death. In addition, normal prostate epithelial cells grown in a medium containing copper become susceptible to isoflavone-induced growth inhibition. We also demonstrate the importance of the copper transporters genes *CTR1* and *ATP7A* in the survival dynamics of malignant cells after exposure to isoflavone. The chemical structures of the genistein and daidzein used in the studies are given in Figure 1.

## 2. Results

### 2.1. Isoflavones Inhibit Growth and Induce Apoptosis in Prostate Cancer Cells

**Figure 2:**
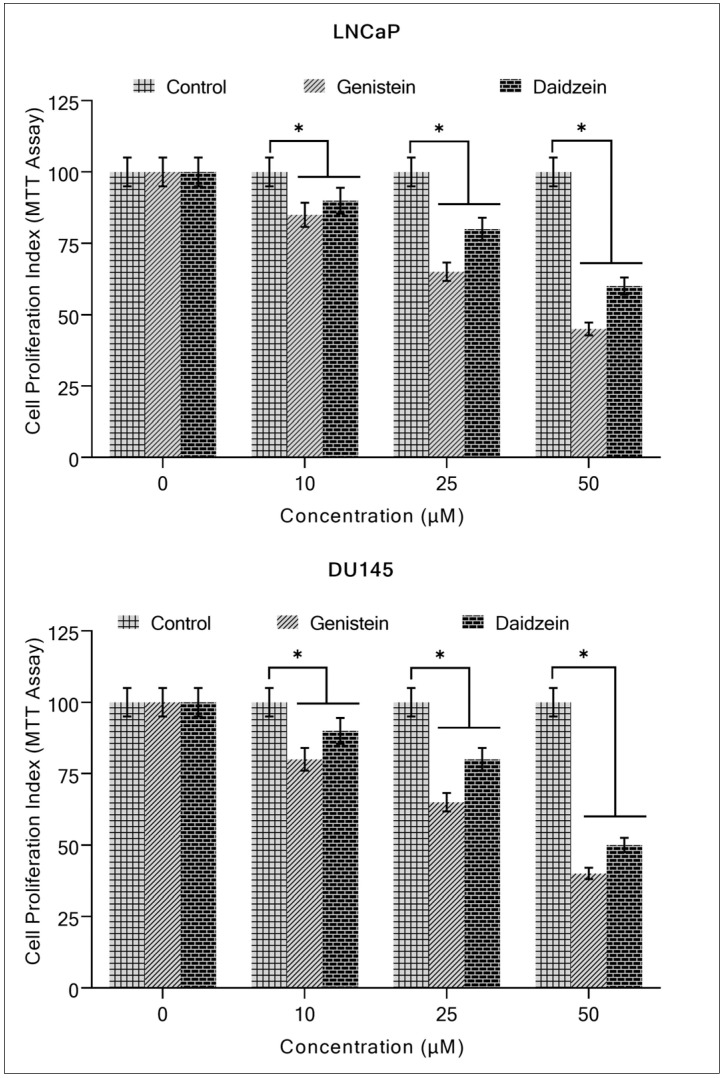
The effect of genistein and daidzein on the proliferation of prostate cancer cell lines determined by the MTT assay. The LNCaP and DU145 cancer cell lines were grown with genistein and daidzein at the given concentrations for 96 h. The effect on cell proliferation was performed by MTT assay as described in Section 4. Values reported are mean ± S.E of triplicate experiments. * *p* < 0.01 compared to the untreated control (0 µM of the isoflavone).

The effects of genistein and daidzein on the proliferation potential of prostate cancer cells LNCaP and DU145 were detected. The MTT assay revealed that the two isoflavones inhibited the growth of these cells in a concentration-dependent manner (Figure 2).

**Figure 3:**
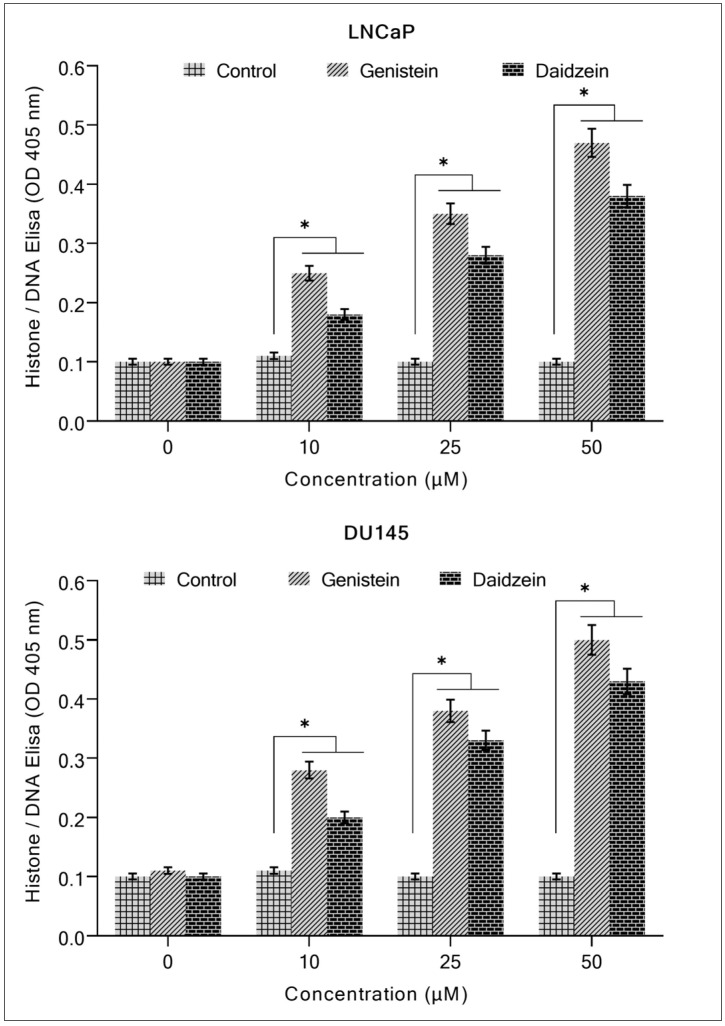
Analysis of genistein and daidzein on apoptosis in prostate cancer cell lines. After incubating prostate cancer cell lines for 96 h with increasing doses of both the isoflavones, apoptosis was detected using the Cell Death Detection ELISA Kit (Roche, Palo Alto, CA, USA), as shown in the figure and discussed in Section 4. Values reported are mean ± S.E of three independent experiments. * *p* value < 0.01 when compared to control.

For further validation of these results, a Histone/DNA ELISA assay was used (Figure 3). This experiment confirmed that both isoflavones (genistein and daidzein) are potent apoptosis inducers and exhibit a dose-dependent cytotoxic effect.

### 2.2. Copper Chelation Decreases Isoflavone-Induced Growth Inhibition and Apoptosis

**Figure 4:**
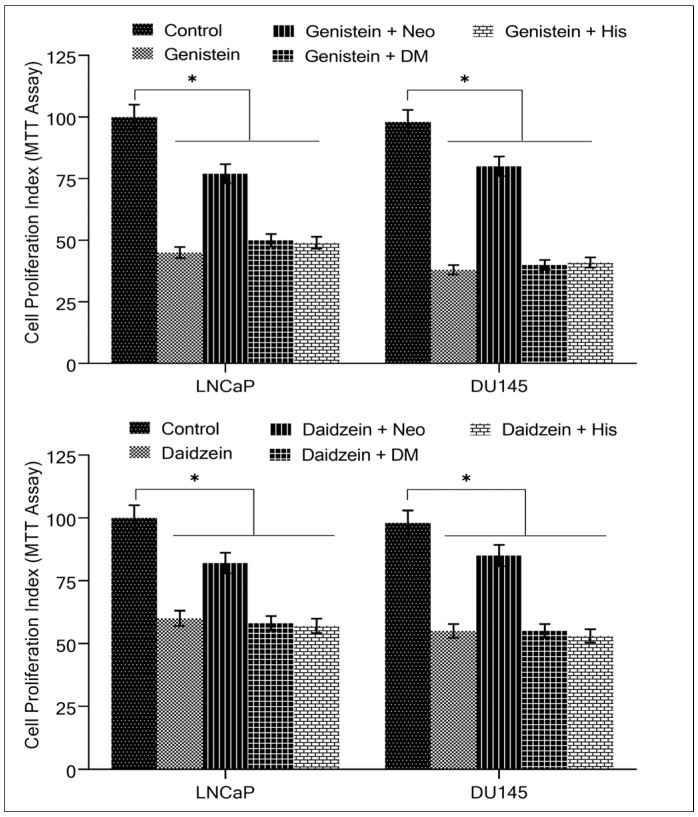
The effect of different metal chelators on the antiproliferative effects of genistein and daidzein in prostate cancer cell lines. As indicated in the figure, LNCaP and DU145 cancer cells were treated with 50 µM genistein/and daidzein either alone or in the presence of copper chelator neocuproine (Neo), iron chelator desferrioxamine mesylate (DM) or zinc chelator histidine (His). Metal chelators were utilized at a concentration of 50 µM. The MTT assay was done 96 h following treatment, as stated in Section 4. Values reported are mean ± S.E of three independent experiments. * *p* value < 0.01 when compared to control.

We previously demonstrated that neocuproine, a membrane-permeable copper chelator, reduces isoflavone-induced oxidative DNA breakage in lymphocytes [33], implying the involvement of intracellular copper in the process. We pondered whether cancer cells were also susceptible to this copper chelator effect. We conducted the MTT assay using the cancer cells LNCaP and DU145 to study the antiproliferative effects of different metal chelators. Figure 4 shows that only the copper chelator neocuproine could considerably protect cells from the growth-inhibiting effects of genistein and daidzein, while desferrioxamine mesylate (an iron chelator) and histidine (a zinc chelator) both had negligible effects. The concentration of the various metal chelators used was 50 µM.

**Figure 5:**
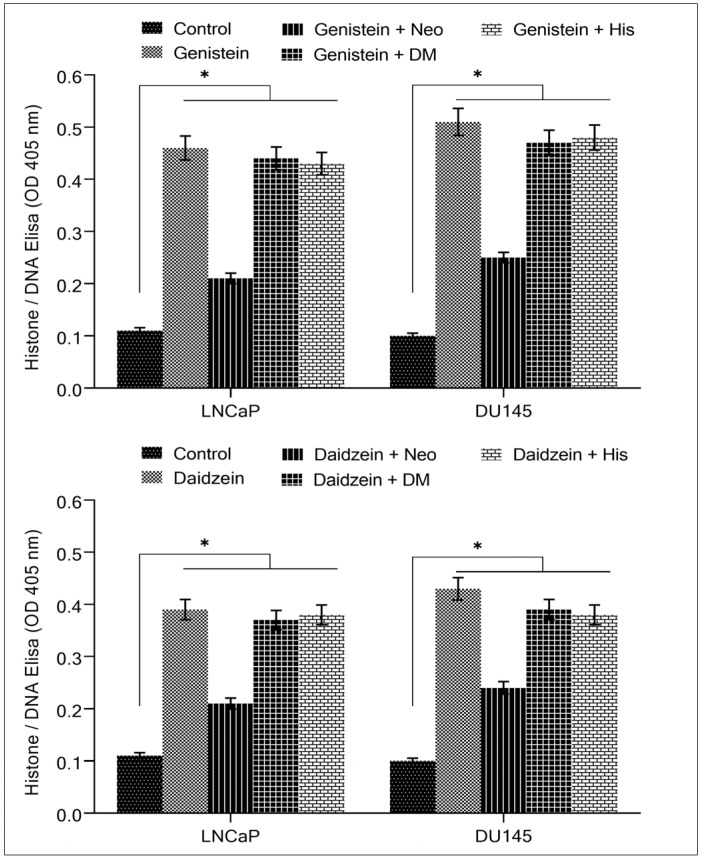
The effect of metal chelators on isoflavone-induced apoptosis in prostate cancer cell lines. LNCaP and DU145 cancer cells were treated with 50 µM genistein/and daidzein alone or in the presence of the copper chelator neocuproine (Neo), iron chelator desferrioxamine mesylate (DM), or zinc chelator histidine (His). The chelators used had a concentration of 50 µM. Apoptosis was detected using the Cell Death Detection ELISA Kit (Roche, Palo Alto, CA, USA). Values reported are mean ± S.E of three independent experiments. * *p* value < 0.01 when compared to control.

To confirm the interesting results obtained in Figure 4, we decided to investigate the capacity of metal chelators to inhibit isoflavone-induced apoptosis. The copper chelator neocuproine provided a considerable reduction of isoflavone on apoptosis induction. That was not seen with iron or zinc chelators. Figure 5 supports the idea that the anticancer mechanism of isoflavones requires the mobilization of copper [24,25,26].

### 2.3. Apoptosis of Cancer Cells Induced by Isoflavones Is Mediated by Reactive Oxygen Species

Reactive oxygen species are generated during DNA breaks by prooxidant anticancer compounds [24,25,26]. The effect of ROS scavengers (thiourea, catalase, and superoxide dismutase) on genistein/and daidzein-induced apoptosis of cancer cells was investigated with the explicit purpose of determining whether isoflavone-induced DNA damage in cancer cell lines involves ROS. In the prostate cancer cell lines studied, all three ROS scavengers exhibited moderate to significant suppression of isoflavone-induced apoptotic activity (Table 1). According to these results, ROS is indeed an important effector of isoflavone-induced apoptosis [34,35], probably via a Fenton-type physiologically active reaction, as has been previously shown [36,37,38].

The effect on apoptosis was evaluated using the Histone/DNA ELISA as described in Section 4. A variety of ROS-scavengers, in addition to isoflavones, were utilized to treat cancer cells, including 700 µM Thiourea, 100 µg/mL Catalase, and 100 µg/mL Superoxide dismutase. The values are mean ± S.E. of three separate experiments. “Apoptosis (folds)” is the fold increase in apoptosis compared to control. The effect of scavengers was calculated using the formula:

$$\mathrm{Effect}\;\mathrm{of}\;\mathrm{scavengers}=\frac{\mathrm{Isoflavone}\;\mathrm{alone}\;-\left(\mathrm{Isoflavone}+\mathrm{ROS}\;\mathrm{scavenger}\right)}{\mathrm{Isoflavone}\;\mathrm{alone}}\times 100$$

### 2.4. Copper Chelation Suppresses Isoflavone-Induced Inhibition of Migration by Cancer Cells

**Figure 6:**
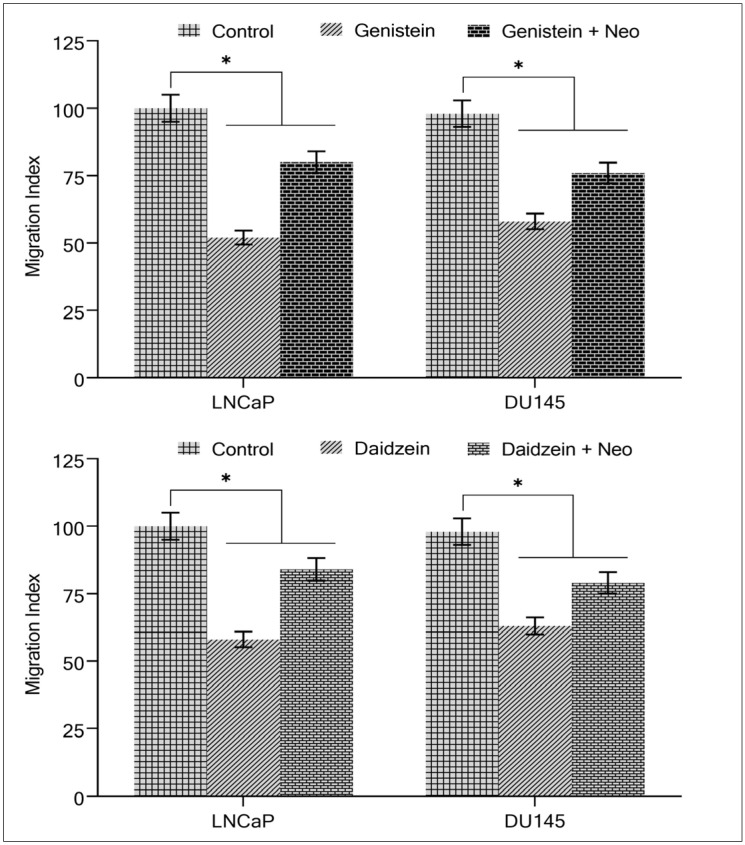
The effect of isoflavones on prostate cancer cell migration in the presence of the copper chelator neocuproine. The assay was carried out as described in Section 4. The cells were cultured with and without genistein/and daidzein (50 µM) and with or without neocuprione (50 µM). Values reported are mean ± S.E. of three independent experiments. * *p* value < 0.01 when compared to control.

It is well-known that metastasis and spreading to new sites is a hallmark of cancer cells. We suggested determining the effect of the migration of prostate cancer cells in the presence of isoflavone with or without a copper chelator. As was expected based on the above results, Figure 6 shows that isoflavone inhibited the capacity of LNCaP and DU145 cells to migrate, making them less likely to metastasize. Interestingly, the malignant cells metastatic potential was significantly restored when copper was chelated from the cells using neocuprione in the presence of genistein/and daidzein (Figure 6), suggesting that cellular copper plays a role in isoflavone-induced inhibition of malignant cell migration.

### 2.5. Copper Supplementation Sensitizes Normal Prostate Epithelial Cells to Antiproliferative Action of Isoflavones

**Figure 7:**
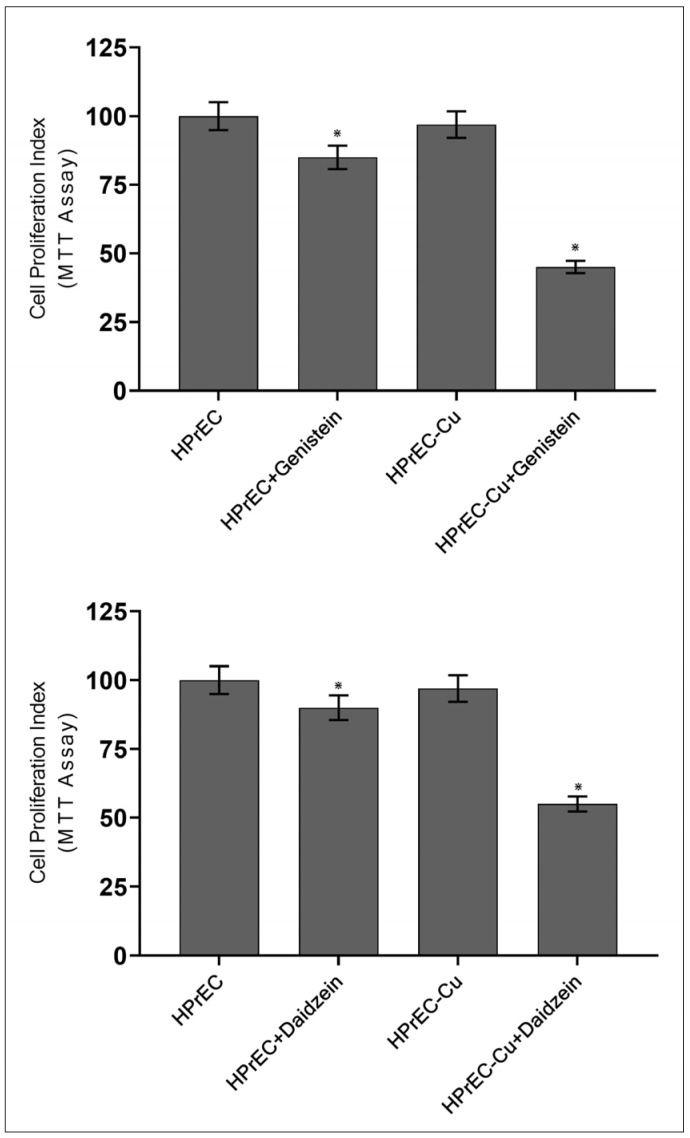
The effect of isoflavones on cell proliferation inhibition in normal prostate epithelial cells (HPrEC) and HPrEC cells cultured in copper-supplemented media (HPrEC-Cu). HPrEC and HPrEC-Cu (normal cells cultured in a medium containing 25 µM CuCl_2_) were treated for 96 h with a 50 µM concentration of genistein/and daidzein. The cell proliferation was then assessed using the MTT assay, as indicated in Section 4. Values reported are mean ± S.E. of three independent experiments. * *p* value < 0.01 when compared to respective control.

To elucidate the importance of copper on cell proliferation in the presence of isoflavones, we used HPrEC (normal (non-malignant) prostate epithelial cells). These cells were grown in a medium containing 25 µM copper. When such copper-supplemented cells (HPrEC-Cu) were treated with genistein/and daidzein, there was a substantial decrease in cell proliferation compared to non-copper-supplemented HPrEC cells (Figure 7).

Since malignant transformation is accompanied by a drastic rise in intracellular copper levels of malignant cells [31,39], it is reasonable to infer that the isoflavone-induced growth inhibition of malignant cells is a consequence of its interaction with cellular copper. Exogenous copper supplementation has made non-malignant epithelial cells more susceptible to isoflavone-induced cell growth suppression (Figure 7).

### 2.6. Isoflavone Suppresses the Expression of Copper Transporters CTR1 and ATP7A in Cancer Cells

**Figure 8:**
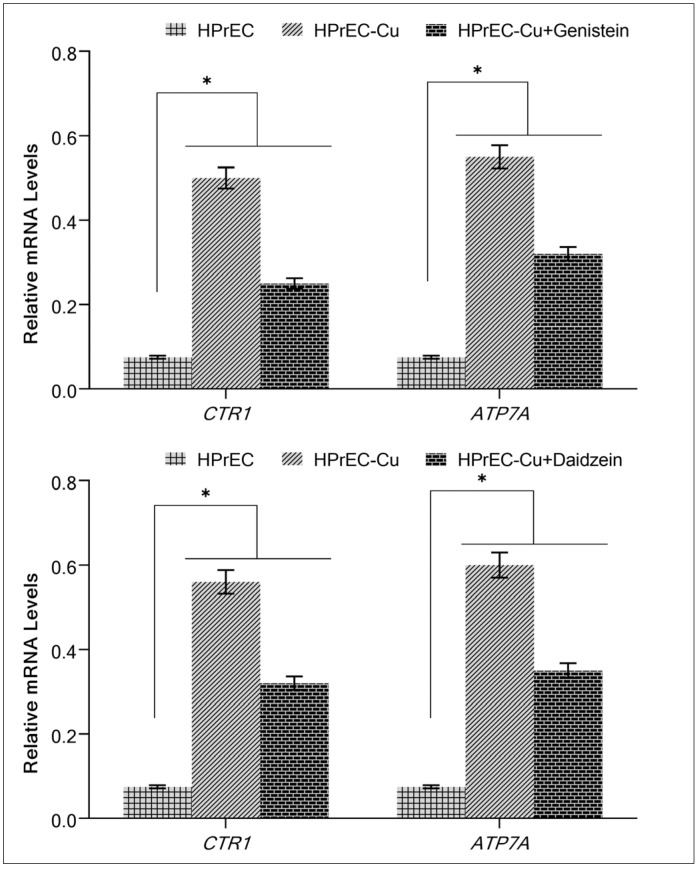
The effect of isoflavones on the increased mRNA levels of copper transporters *CTR1* and *ATP7A* in HPrEC-Cu cells relative to parental HPrEC cells. As mentioned in Section 4, *CTR1* and *ATP7A* mRNA expression was measured using real-time PCR. Only HPrEC-Cu cells (regular HPrEC cells cultured in a medium containing 25 µM CuCl_2_) with elevated mRNA expression of copper transporters were treated with genistein/and daidzein (50 µM). Values reported are mean ± S.E. of three independent experiments. * *p* value < 0.01 when compared to control.

Isoflavone inhibits growth in malignant cells (Figure 4 and Figure 5) and non-malignant epithelial cells cultivated in copper-supplemented media, as shown by the interaction between the isoflavone and intracellular copper (Figure 7). Due to the higher levels of *CTR1* and *ATP7A* produced by malignant cells [39], we have investigated whether copper supplementation increases copper transporter expression in epithelial cells that are not malignant. Expression of the copper transporters *CTR1* and *ATP7A* was significantly upregulated when copper was added to the HPrEC cell growth medium (Figure 8). The expression of both copper transporters was significantly suppressed upon further addition of genistein/and daidzein to the medium (Figure 8), suggesting an action of isoflavones on copper metabolism in cancer cells.

### 2.7. Silencing of CTR1 and ATP7A in HPrEC Cells Grown in Copper-Supplemented Medium Reduces Isoflavone-Induced Inhibition of Proliferation

**Figure 9:**
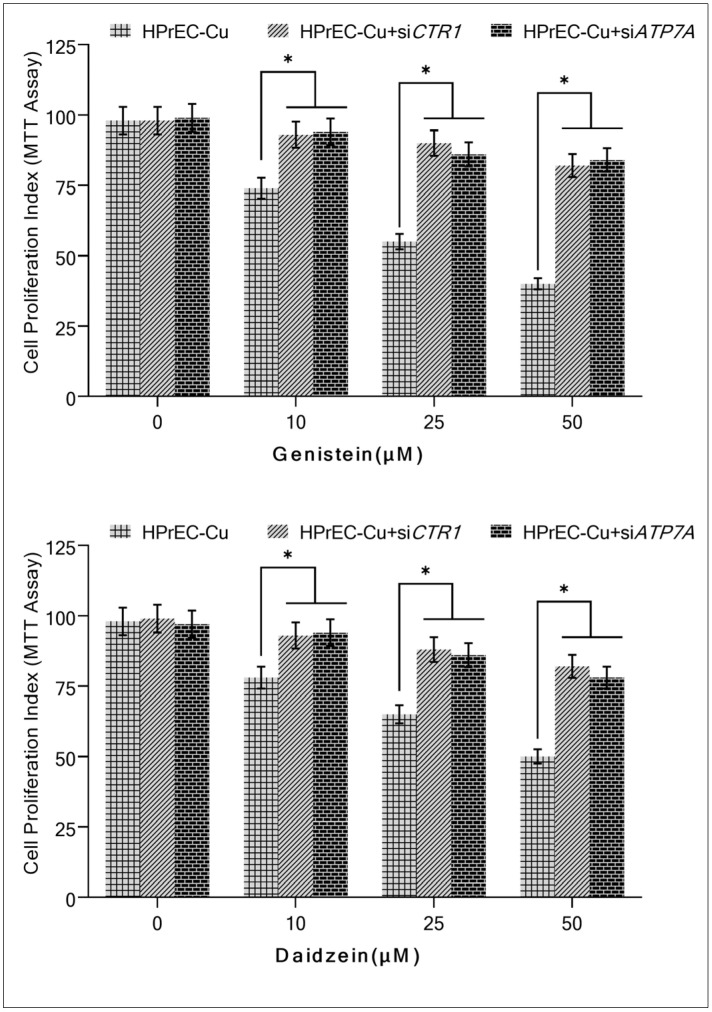
The effect of isoflavones on cell proliferation of HPrEC-Cu cells (normal HPrEC cells cultured in a medium containing 25 µM CuCl_2_) was compromised after the knock-down of *CTR1* and *ATP7A*. HPrEC-Cu cells were initially treated for 48 h with targeted siRNA against *CTR1* (*siCTR1*) and *ATP7A* (*siATP7A*), followed by 96 h with the indicated doses of genistein/and daidzein. Values reported are mean ± S.E. of three independent experiments. * *p* value < 0.01 when compared to respective control.

To further substantiate the role of copper in isoflavone-induced growth suppression, we silenced the copper transporters *CTR1* and *ATP7A* with targeted siRNA (Figure 9). As shown in Figure 8, HPrEC cells are more sensitive to isoflavone-induced growth suppression (inhibition of cell proliferation) when *CTR1* and *ATP7A* are expressed. CTR1 and ATP7A proteins promote copper uptake in cells [40]. The silencing of the copper transporters *CTR1*/*ATP7A* compromised the sensitivity of HPrEC cells to genistein/and daidzein in the copper-enriched medium. Such evidence conclusively demonstrates that isoflavones interact with cellular copper, which is required for the isoflavone’s growth-suppressing effect against cancer cells.

This provides conclusive evidence that isoflavones affect intracellular copper and that this interaction is required for isoflavones to prevent the proliferation of cancer cells.

## 3. Discussion

Over the past years, our research group has devoted much attention to investigating oxidative DNA-breaking processes mediated by polyphenolic compounds while copper ions are present [41,42,43,44,45,46]. The most important findings are as follows, based on the results presented above (a) The proliferation of human prostate cancer cell lines is suppressed by isoflavones in a concentration-dependent order. Genistein/and daidzein inhibited cancer cell growth, leading to death in such cells, although the copper chelator neocuproine reversed this effect. This finding demonstrates the role of copper in the cytotoxicity caused by isoflavones. (b) Additionally, copper redox cycling in the presence of the isoflavones examined generates ROS, as evidenced by a reduction in apoptosis induction when ROS scavengers are present; this implicates copper as a molecular target for the cancer-cell-inhibitory effect of isoflavones. (c) According to our findings, daidzein, which differs structurally from genistein only by the absence of a hydroxyl group (OH) in position C-5, has slightly lesser inhibitory effects in human prostate cancer cells. It has also previously been observed with different polyphenols that the number of hydroxyl substitutions in a flavonoid’s backbone structure influences its prooxidant and antioxidant properties [33,45,46,47]. Our findings are consistent with these observations, and we conclude that hydroxyl groups are critical components of isoflavone biological activity.

It is exciting to see that normal prostate epithelial HPrEC cells are more resistant to the cytotoxicity caused by isoflavones than malignant prostate cells. This data demonstrates the selectivity of the isoflavone cytotoxicity in cancer cells [26,34]. Our research has shown that when HPrEC cells are cultivated in the presence of copper, they become more susceptible to the cytotoxicity caused by isoflavone. This provides more evidence that copper plays an essential role in the physiological reactions mediated by isoflavones and ultimately ends in cell death [26]. While Fe^3+^ and Cu^2+^ are the most redox-active metal ions in living cells, it has been established in multiple studies that only copper is considerably raised in the cancer cells of patients [27,28,29,30,31]. Previous research has demonstrated that normal breast epithelial MCF10A cells had no detectable copper [34], which may account for their resistance to isoflavone in the present study.

Copper’s physiological significance in cancer is only vaguely known at this point. Despite this, there are plenty of available pieces of evidence showing that elevated copper levels contribute to tumor angiogenesis [26,48]. Our experimental hypothesis [24,25] suggests that plant polyphenols interact with copper within the cell and trigger oxidative DNA breakage has recently been confirmed by using a wide range of experiments and a variety of polyphenols [40,49,50,51,52]. The results of the current investigation provide even more support for the ideas we have generated as a result of our prior research.

When normal prostate epithelial cells were grown in the presence of copper, it was shown that the expression of both *CTR1* and *ATP7A*, the two copper transporters genes investigated in this work, was upregulated. The expression of these transporter genes was suppressed by genistein/and daidzein. This observation supports our theory that isoflavones interfere with copper metabolism in transformed cells by inhibiting copper transporters in addition to interacting with copper and causing oxidative DNA damage, as was confirmed in prior research [26,31,39,48].

To better understand the significance of copper transporters genes *CTR1* and *ATP7A*, we used siRNA (Figure 9) to silence the representative copper transporters investigated in this study. In HPrEC cultures supplemented with copper, inhibiting *CTR1*/and *ATP7A* led to a reduction in the sensitivity of the cells to isoflavone. This finding established and verified that copper is necessary for isoflavone-mediated selective cell death.

Evidence shows that those whose diets include soy have a reduced cancer rate [33,34]. Soy products were found to significantly protect against death from prostate cancer in a major investigation conducted in 1998 & 2014, respectively [53,54]. Despite promising epidemiological and preclinical data, it is difficult to draw definitive conclusions regarding the clinical efficacy of isoflavones.

It has been noted that the average daily intake of isoflavones is 15–60 mg in Asian nations with high soy and soy-derived food consumption [55,56], but only 1–2 mg in Western countries [57,58]. After digestion and excretion, the plasma level of genistein in soy-rich diet eaters is only 1–5 mM [34]. The bioavailability of isoflavones is a topic that requires more research because its mechanisms and the molecules involved in the signaling pathways that cause the death of cancerous cells are still a mystery and need to be investigated in future research. Further, genistein/or daidzein is only one of the polyphenols consumed in the diet. The combined concentration, bioavailability, and action of polyphenols, such as flavonoids, tannins, etc., may be greater than those of dietary isoflavone alone, as all polyphenols are active prooxidants [26,59,60,61,62]. Nonetheless, it is important to note that different dietary polyphenols have a relatively limited half-life in vivo since they are rapidly digested [60,61,62]. For this reason, it is possible that plant polyphenols are not as effective as anticancer medications already in use in clinical settings. In this sense, establishing plant polyphenols as powerful lead compounds to manufacture novel anticancer medicines would benefit from identifying a definitive anticancer mechanism.

## 4. Materials and Methods

### 4.1. Materials

Genistein, daidzein, DMSO, phosphate-buffered saline (PBS) Ca^2+^ and Mg^2+^, RPMI, cupric chloride (purity ≥ 99%), metal chelators: neocuproine, desferrioxamine mesylate, and histidine were acquired from Sigma Chemical Co. (St. Louis, MO, USA). All additional compounds were of analytical grade and purchased from commercial suppliers.

### 4.2. Cell Lines and Reagents

The immortalized non-transformed prostate cell line HPrEC and the prostate cancer cell lines LNCaP and DU145 were obtained from ATCC (Manassas, VA, USA) and maintained following the protocol as described previously [40]. The stock solutions of genistein and daidzein were prepared in DMSO, whereas the stock solutions of metal chelators were prepared in PBS. A normal prostate epithelial cell line, HPrEC, was propagated using the protocol described earlier [40]. HPrEC-Cu cells are HPrEC cells that were cultivated in their standard culture media with additional supplementation of CuCl_2_ (25 µM) for thirty days.

### 4.3. Cell Growth Inhibition Studies by 3-(4,5-Dimethylthiazol-2-yl)-2,5 Diphenyl-Tetra-Zolium Bromide (MTT) Assay

The MTT experiment was carried out exactly as stated previously [40]. LNCaP and DU145 cells were seeded at a density of 2 × 10^3^ cells per well on 96-well microtiter culture plates. Cells were exposed to the mentioned isoflavone concentrations after overnight incubation. Metal chelators were used in individual assays, as mentioned in their specific experiments. Each treatment contained eight replicate wells, and the amount of DMSO in the reaction mixture was never more than 0.1%. Furthermore, each experiment was performed three times.

### 4.4. Histone/DNA ELISA for Detection of Apoptosis

Following the manufacturer’s instructions, the cell death detection ELISA Kit (Roche, Palo Alto, CA, USA) was used to detect apoptosis in LNCaP and DU145 prostate cancer cells treated with isoflavones, following the protocol reported as previously described [40]. At the concentrations indicated, metal chelators and ROS scavengers were added.

### 4.5. Cell Migration Assay

The migration of cells was measured using 24-well transwell permeable supports with 8-mm pores (Corning), as previously described [40]. Using ULTRA Multifunctional Micro-plate Peruser (TECAN), we analyzed the migrated cells by reading their fluorescence. Cells were cultured at the indicated concentrations with and without neocuprione and isoflavones.

### 4.6. Real-Time Reverse Transcriptase PCR

Total RNA was extracted using the TRIzol (Invitrogen, Carlsbad, CA, USA) reagent according to the manufacturer’s instructions. Real-time PCR was used to measure mRNA expression. The primer sequences for *CTR1* (forward: 5′-GCT GGA AGA AGG CAG TGG TA-3′; reverse: 5′-AAA GAG GAG CAA GAA GGG ATG-3′), *ATP7A* (forward: 5′-ACG AAT GAG CCG TTG GTA GTA-3′; reverse: 5′-CCT CCT TGT CTT GAA CTG GTG-3′) and *GAPDH* (glyceraldehyde-3-phosphate dehydrogenase) (forward: 5′-TGG GTG TGA ACC ATG AGA AGT-3′; reverse: 5′-TGA GTC CTT CCA CGA TAC CAA-3′) were the same as previously reported [40,63], and the amount of RNA was normalized to the housekeeping gene *GAPDH* expression.

### 4.7. siRNA (Small Interfering RNA) Transfection

siRNA transfections were carried out as previously described [40]. Santa Cruz Biotechnology, Inc. (Santa Cruz, Dallas, TX, USA). provided siRNA for *CTR1* and *ATP7A*. Scrambled siRNA was employed as a nonspecific control. Transfections were carried out using Lipofectamine RNA iMAX Transfection Reagent (Invitrogen) according to the manufacturer’s instructions. *CTR1* and *ATP7A* were silenced by siRNA for 48 h before the experiment.

### 4.8. Statistical Analysis

The results are expressed as the mean ± S.E. of three independent experiments. The Student’s t-test was performed to look for statistically significant differences. ANOVA was used for variance analysis. *p*-values < 0.05 were considered statistically significant.

## 5. Conclusions

**Figure 10:**
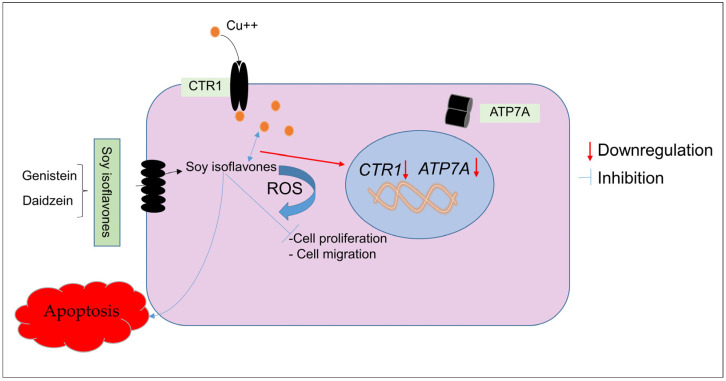
A proposed schematic diagram showing the interaction of isoflavones and copper in the down-regulation of *CTR1* and *ATP7A*. In addition, the involvement of redox cycling generates reactive oxygen species, leading to DNA damage and, ultimately, apoptosis.

Overall, the results of our study are consistent with the notion that the oxidative DNA damage caused by isoflavones is significantly influenced by the internal copper concentration in cancer cells (Figure 10). In addition to providing new insight into how isoflavones function as chemopreventive agents, the above findings also contribute to a general explanation of why many polyphenolic compounds with various chemical structures have anticancer activities. This paves the way for later mechanism-based research and clinical trials that will concentrate on focusing on the microenvironment of tumors to achieve the necessary degree of efficacy of non-toxic anticancer drugs employing naturally occurring plant-based polyphenols.

## Figures and Tables

**Table 1:** Effect of ROS scavengers on isoflavone activity in prostate cancer cells.

Cancer Cell Line	Dose	Apoptosis (Folds)	Effect of Scavengers
**LNCaP**	Untreated	-	-
	Genistein (50 µM)	4.42 *	-
	Thiourea	2.48 *	43.89
	Catalase	3.15 *	28.73
	Superoxide dismutase	3.43 *	22.39
**LNCaP**	Untreated	-	-
	Daidzein (50 µM)	4.62 *	-
	Thiourea	2.86 *	38.09
	Catalase	3.38 *	26.83
	Superoxide dismutase	3.64 *	21.21
**DU145**	Untreated	-	-
	Genistein (50 µM)	5.81 *	-
	Thiourea	3.79 *	34.76
	Catalase	4.23 *	27.19
	Superoxide dismutase	4.55 *	21.68
**DU145**	Untreated	-	-
	Daidzein (50 µM)	5.34 *	-
	Thiourea	3.48 *	34.83
	Catalase	3.73 *	30.14
	Superoxide dismutase	4.06 *	23.97

* *p* value < 0.05 when compared to respective control.

## Data Availability

Not applicable.
